# Impaired *Mycobacterium tuberculosis*-specific T-cell memory phenotypes and functional profiles among adults with type 2 diabetes mellitus in Uganda

**DOI:** 10.3389/fimmu.2024.1480739

**Published:** 2024-10-04

**Authors:** Phillip Ssekamatte, Rose Nabatanzi, Diana Sitenda, Marjorie Nakibuule, Bernard Ssentalo Bagaya, Davis Kibirige, Andrew Peter Kyazze, David Patrick Kateete, Obondo James Sande, Reinout van Crevel, Stephen Cose, Irene Andia Biraro

**Affiliations:** ^1^ Department of Immunology and Molecular Biology, School of Biomedical Sciences, College of Health Sciences, Makerere University, Kampala, Uganda; ^2^ Medical Research Council/Uganda Virus Research Institute and London School of Hygiene & Tropical Medicine, Entebbe, Uganda; ^3^ Department of Medicine, Uganda Martyrs Lubaga Hospital, Kampala, Uganda; ^4^ Department of Internal Medicine, School of Medicine, College of Health Sciences, Makerere University, Kampala, Uganda; ^5^ Department of Internal Medicine and Radboud Centre for Infectious Diseases, Radboud University Medical Centre, Nijmegen, Netherlands

**Keywords:** latent tuberculosis infection, diabetes mellitus, T cells, memory phenotypes, functional profiles

## Abstract

**Background:**

Efforts to eradicate tuberculosis (TB) are threatened by diabetes mellitus (DM), which confers a 3-fold increase in the risk of TB disease. The changes in the memory phenotypes and functional profiles of *Mycobacterium tuberculosis* (*Mtb*)-specific T cells in latent TB infection (LTBI)-DM participants remain poorly characterised. We, therefore, assessed the effect of DM on T-cell phenotype and function in LTBI and DM clinical groups.

**Methods:**

We compared the memory phenotypes and function profiles of *Mtb*-specific CD4^+^ and CD8^+^ T cells among participants with LTBI-DM (n=21), LTBI-only (n=17) and DM-only (n=16). Peripheral blood mononuclear cells (PBMCs) were stimulated with early secretory antigenic 6 kDa (ESAT-6) and culture filtrate protein 10 (CFP-10) peptide pools or phytohemagglutinin (PHA). The memory phenotypes (CCR7/CD45RA), and functional profiles (HLA-DR, PD-1, CD107a, IFN-γ, IL-2, TNF, IL-13, IL-17A) of *Mtb*-specific CD4^+^ and CD8^+^ T cells were characterised by flow cytometry.

**Results:**

Naïve CD4^+^ T cells were significantly decreased in the LTBI-DM compared to the LTBI-only participants [0.47 (0.34-0.69) vs 0.91 (0.59-1.05); (p<0.001)]. Similarly, CD8^+^ HLA-DR expression was significantly decreased in LTBI-DM compared to LTBI-only participants [0.26 (0.19-0.33) vs 0.52 (0.40-0.64); (p<0.0001)], whereas CD4^+^ and CD8^+^ PD-1 expression was significantly upregulated in the LTBI-DM compared to the LTBI-only participants [0.61 (0.53-0.77) vs 0.19 (0.10-0.28); (p<0.0001) and 0.41 (0.37-0.56) vs 0.29 (0.17-0.42); (p=0.007)] respectively. CD4^+^ and CD8^+^ IFN-γ production was significantly decreased in the LTBI-DM compared to the LTBI-only participants [0.28 (0.19-0.38) vs 0.39 (0.25-0.53); (p=0.030) and 0.36 (0.27-0.49) vs 0.55 (0.41-0.88); (p=0.016)] respectively. CD4^+^ TNF and CD8^+^ IL-17A production were significantly decreased in participants with LTBI-DM compared to those with LTBI-only [0.38 (0.33-0.50) vs 0.62 (0.46-0.87); (p=0.004) and 0.29 (0.16-0.42) vs 0.47 (0.29-0.52); (0.017)] respectively. LTBI-DM participants had significantly lower dual-functional (IFN-γ^+^IL-2^+^ and IL-2^+^TNF^+^) and mono-functional (IFN-γ^+^ and TNF^+^) CD4^+^ responses than LTBI-only participants. LTBI-DM participants had significantly decreased dual-functional (IFN-γ^+^IL-2^+^, IFN-γ^+^ TNF^+^ and IL-2^+^TNF^+^) and mono-functional (IFN-γ^+^, IL-2^+^ and TNF^+^) central and effector memory CD4^+^ responses compared to LTBI-only participants.

**Conclusion:**

Type 2 DM impairs the memory phenotypes and functional profiles of *Mtb*-specific CD4^+^ and CD8^+^ T cells, potentially indicating underlying immunopathology towards increased active TB disease risk.

## Introduction

Despite significant efforts made to control tuberculosis (TB), the increasing burden of diabetes mellitus (DM) threatens the progress registered in reducing the global burden of TB, especially in low and middle-income countries (LMICs) (1). According to the 2021 International Diabetes Federation (IDF) estimates, approximately 537 million adults (aged between 20 and 79) live with DM. This figure is projected to rise to 783 million by 2045, with the most significant increase in Africa (2). Tuberculosis remains one of the leading causes of death from a single infectious agent, *Mtb*, worldwide (3). Globally, approximately 7.5 million people were newly infected with *Mycobacterium tuberculosis* (*Mtb*) or diagnosed with TB in 2022, with nearly 1.3 million deaths occurring (3). Epidemiologically, DM confers a 3-fold increase in the risk of developing TB disease and is associated with TB treatment failure and drug resistance (4). Indeed, it was recently reported that participants aged ≥ 40 years had increased odds of TB-DM comorbidity (5) and that Africans with DM have an increased latent TB infection (LTBI) risk (6). The risk for the development of active TB (ATB) is thought to be due to the immune-compromised status, but the underlying susceptibility mechanisms remain largely unknown.

The quality of the T-cell response is essential for *Mtb* immunity. CD4^+^ and CD8^+^ T cells are pivotal for immune control in *Mtb*-infected humans and murine TB models (7, 8). T-cell memory phenotypes are induced during LTBI and Bacillus Calmette-Guerin (BCG) vaccination that play a protective role in humans and in mice models (9–12). It is reported that LTBI is characterised by differential expression of functional markers, including decreased HLA-DR expression, a marker that distinguishes LTBI and ATB (13, 14), upregulated PD-1 expression, a marker that inhibits T-cell effector functions (15, 16), as well as downregulated Th1 (7) and Th17 (17, 18) cytokine production. Examining cytokine T-cell polyfunctionality is essential as these cells have been associated with resistance to infection (19, 20). Elevated frequencies of mono-functional and dual-functional CD4^+^ Th1 cells are reportedly a hallmark of active TB and DM (TB-DM) comorbidity (21). This shows that type 2 DM modulates T-cell immune responses to *Mtb*, which could profoundly affect TB pathogenesis. However, the underlying immunological mechanisms for TB susceptibility during DM remain to be elucidated, specifically with phenotypes and functional markers during LTBI.

In this study, we hypothesised that type 2 DM modulates the *Mtb*-specific memory phenotype and functional profiles of T cells among participants with LTBI, leading to impaired responses and potentially promoting TB susceptibility, progression or reactivation. We aimed to assess the *Mtb*-specific CD4^+^ and CD8^+^ T-cell memory phenotypes and functional profiles. We compared the T-cell memory, activation, degranulation, exhaustion and cytokine polyfunctionality profiles among participants with LTBI-DM comorbidity.

## Materials and methods

### Study population and setting

Participants with LTBI and DM (LTBI-DM) and DM-only participants were enrolled from October 2018 to March 2019 at the DM clinic at Kiruddu National Referral Hospital. This was part of the Tuberculosis and Diabetes (TAD) study (22), a longitudinal study which explored isoniazid prophylaxis outcomes among DM participants with LTBI and ATB. Participants with LTBI-only were enrolled in a TB household contact cohort [Kampala TB (KTB)] study from May 2011 to January 2012, Kampala, Uganda, at Kisenyi and Kitebi Health Centre IVs, as previously described (23). To get a proper negative control group, the study utilised LTBI-only PBMC samples from the KTB study, which did not collect DM-related parameters [weight, random blood sugar (RBS), blood pressure and HbA1c]. While LTBI-DM and LTBI-only are the main comparator groups, the DM-only group was included as a negative control to compare and assess how DM alone (without LTBI) might impact immune function.

### Study methods

Peripheral blood mononuclear cell samples taken from 54 participants were assayed using flow cytometry (LTBI-DM [n=21], LTBI-only [n=17] and DM-only [n=16]). Diabetes Mellitus was diagnosed based on the American Diabetes Association (ADA) criteria (glycated haemoglobin [HbA1c] levels ≥ 6.5%), with normal ranges between 4% and 5.6% (24). Latent TB infection was diagnosed based on positive results for QuantiFERON TB-Gold (QFT)-Plus and QFT In-Tube assays. All participants were adults and HIV-negative.

### Peripheral blood mononuclear cell isolation

Ten millilitres of heparinised blood collected by venepuncture was transported within 4 hours to the immunology laboratory at the College of Health Sciences, Makerere University and the MRC/UVRI and LSHTM Uganda Research Unit, Kampala, Uganda, for processing. Peripheral blood mononuclear cells (PBMCs) were isolated using Ficoll-Histopaque density gradient centrifugation. The Cells were counted and resuspended in cold foetal bovine serum (FBS) supplemented with 10% dimethyl sulfoxide (DMSO). Cells were then adjusted to a final concentration of 3x10^6^ cells/ml. Cells were transferred to a cold Mr Frosty™ freezing container overnight at -80°C and then moved to liquid nitrogen (-197°C) for long-term storage.

### Cell stimulation and culture

Upon retrieval from liquid nitrogen, frozen cell vials (6x10^6^ cells) were thawed at a 37°C water bath in R20 (RPMI with 20% FBS, 1% Penicillin/streptomycin, 2mM Glutamine, 25mM HEPES). The PBMCs were rinsed and rested in R10 (RPMI with 10% FBS, 1% Penicillin/streptomycin, 2mM Glutamine, 25mM HEPES) media in a humidified incubator at 5%CO_2_, 37°C for 4 hours. The cells (200µl/2x10^6^, resuspended in R20) were stimulated in a humidified incubator at 37°C, 5%CO_2_ for 18 hours (overnight) with *Mtb*-specific peptide pools of early secreted antigenic target-6 kDa [ESAT-6 (21-peptide array; 10µg/ml)], and culture filtrate protein-10 kDa [CFP-10 (22-peptide array; 10µg/ml)], all from BEI Resources (Manassas, VA). The peptides consist of 15- or 16-mers peptides (overlapping by 11 or 12 amino acids) spanning the entire amino acid sequences for the ESAT-6 and CFP-10. Phytohemagglutinin-lectin (PHA-L [10µg/ml, Millipore, Sigma]) was used as a positive control, and unstimulated cells (R20 media) as a negative control. Stimulations were performed for 2 hours, after which Brefeldin A (5µg/ml, BioLegend) was added to all tubes. Cells were further incubated and stimulated for 16 hours. All experiments were performed in the presence of co-stimulatory antibodies, anti-CD28 and anti-CD49d (1µg/ml each, BD Biosciences) and CD107a brilliant violet (BV) 605 (H4A3, BioLegend) antibody for the 18 hours.

### Cell staining

After stimulations, cells were washed with Dulbecco’s phosphate buffered saline (PBS [1X, Sigma-Aldrich]), followed by staining with a fixable viability dye, zombie aqua (BioLegend) at room temperature for 20 minutes in the dark. Cells were then washed with cell staining buffer (BioLegend), blocked for Fcγ receptors using BD Fc block (2.5µg/ml, BD Biosciences) at room temperature for 10 minutes in the dark. Cells were surface stained at 4°C for 30 minutes in the dark with the following antibodies: CD3 FITC (UCHT1; BioLegend), CD4 PerCP-Cyanine5.5 (A161A1; BioLegend), CD8 BV650 (SK1; BioLegend), CCR7 PE-CF594 (2-L1-A; BD Biosciences), PD-1 BV785 (EH12.2H7; BioLegend), HLA-DR PE-Fire 640 (L243; BioLegend), and CD45RA APC-Cy7 (HI100; BioLegend). For intracellular cytokine staining, cells were washed, fixed using fixation buffer (4% paraformaldehyde, BioLegend), and permeabilised using working strength intracellular staining permeabilisation wash buffer (1X, BioLegend) according to manufacturer’s recommendations. Fixed cells were intracellularly stained at room temperature for 20 minutes in the dark with the following antibodies: IFN-γ PE/Cy7 (4S.B3; BioLegend), TNF APC (MAb11; BioLegend), IL-2 PE (MQ1-17H12; BioLegend), BCL-2 BV421 (100; BioLegend), IL-17A APC-R700 (N49-653; BD Biosciences) and IL-13 Alexa Fluor (AF) 350 (32116; R&D Systems). The cells were immediately acquired on the CytoFLEX LX flow cytometer (Beckman Coulter). The flow cytometry antibody panel, including clone and catalogue number, is shown in **Supplementary Table S1**.

### Data and statistical analysis

The flow cytometry data from this study was normalised to minimise batch effects across the two study PBMC T-cell responses using the ComBat algorithm from the “sva” package. The data was then analysed using FlowJo v.10.10.0 (BD Biosciences, San Jose, CA, USA) for Mac. Gating was standardised and set using Fluorescence Minus One (FMO) and compensation controls to correct for spectral overlap. Boolean combination gating was used to calculate frequencies corresponding to seven different combinations of cytokines, including IL-2, TNF and IFN-γ. The gating strategy is shown in **Supplementary Figure S1**. The data was Arcsine transformed, and a linear regression model was fitted with age as a covariate in all groups using R(v.4.4.0). The linear regression results are reported in **Supplementary Table S2**. Statistical tests were performed using GraphPad Prism (v.10.1.1; GraphPad Software, La Jolla, CA, USA). To compare the memory phenotypes and functional profiles of *Mtb*-specific CD4^+^ and CD8^+^ T cells between participant groups, we used the Kruskal–Wallis with Dunn’s tests for multiple comparisons for more than two participant groups. Mann-Whitney U test was used for two-group comparisons. The data was reported after background (unstimulated) subtraction. Unless otherwise stated, all data were reported for ESAT-6 and CFP-10 peptide stimulations. A p-value <0.05 was considered statistically significant.

## Results

### Baseline characteristics of the study participants

The baseline demographic and clinical characteristics of the study participants are summarised in **Table 1**. Age (p<0.0001) and systolic blood pressure (p=0.037) were statistically different between the study participants. Particularly, LTBI-only [24 (24–32)] participants had a lower median age compared to LTBI-DM [50 (47-56)] and DM [48 (39-54)] participants.

**Table 1:** Baseline characteristics of study participants.

	Overall (n=54)	LTBI-DM (n=21)	LTBI (n=17)	DM (n=16)	p-value
Age, years (median [IQR])	43 (30-52)	50 (47-56)	24 (24-32)	48 (39-54)	<0.0001
Sex, n					0.287
Female (%)	35 (64.8)	11 (52.4)	13 (76.5)	11 (68.8)	
Male (%)	19 (35.2)	10 (47.6)	4 (23.5)	5 (31.2)	
Weight, Kg (median [IQR])*	71.8 (61.3- 87.3)	68.0 (58.2- 82.2)		75.2 (63.0- 91.2)	0.464
RBS, mmol/L (median [IQR])*	7.3 (3.5-13.1)	6.6 (0.0-9.0)		9.4 (5.5-14.2)	0.147
Systolic blood pressure, mm Hg (median [IQR])*	134 (125- 151)	147 (127-171)		129 (120- 138)	0.037
Diastolic blood pressure, mm Hg (median [IQR])*	83 (75-95)	90 (76-103)		81 (72- 85)	0.156
HbA1c, % (median [IQR])*	7.0 (6.0-9.1)	7.3 (6.2-9.1)		6.6 (5.5-9.3)	0.308

*Missing in the LTBI-only group.

#### Type 2 DM alters the memory phenotype of *Mtb*-specific CD4^+^ and CD8^+^ T cells

**Figure 1:**
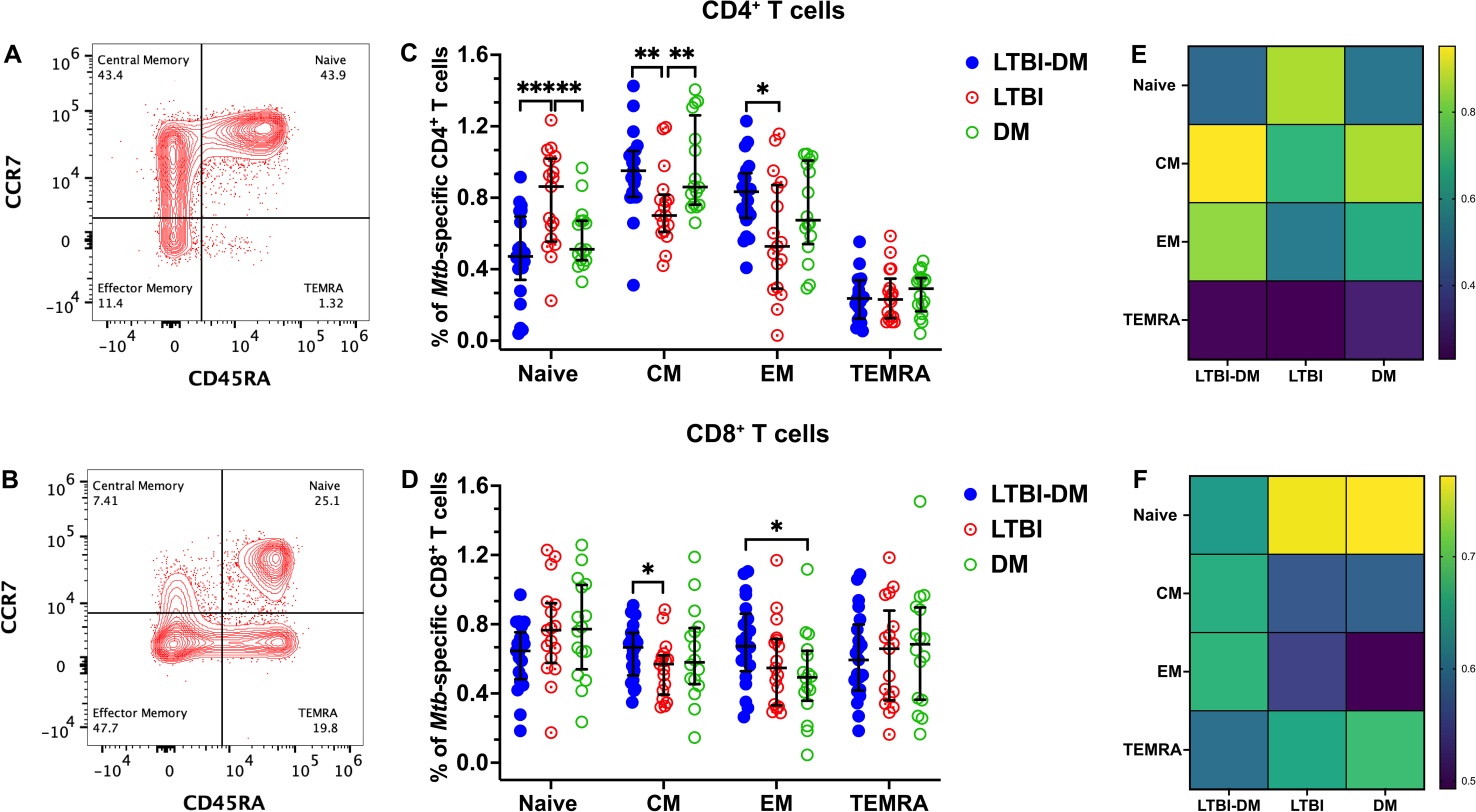
Type 2 DM alters the memory phenotype of *Mtb*-specific CD4^+^ and CD8^+^ T cells. **(A, B)** Representative flow cytometry plots are shown for CD4^+^ and CD8^+^ CCR7/CD45RA-defined T-cell memory subsets, respectively. PBMCs were stimulated and cultured for 18 hours with ESAT-6 and CFP-10 peptide pools plus brefeldin A and stained for surface markers. **(C, D)** Percentage expression of memory phenotypes in CD4^+^ and CD8^+^T cells, respectively. **(E, F)** Heat maps for the percentage distribution of all memory phenotypes in the three CD4^+^ and CD8^+^ T cell participant groups. Size of participant groups: LTBI-DM (n = 21), LTBI (n = 17), DM (n = 16). Data represent medians and interquartile ranges. The non-parametric Kruskal-Wallis and Mann-Whitney U tests were used to determine the statistical significance between the medians. p<0.05 (*), p< 0.01 (**), p<0.001 (***). Non-significant p-values were not shown.

We performed a memory phenotypic analysis of CD4^+^ and CD8^+^ T-cell subsets in participant PBMC samples with LTBI-DM, LTBI-only and DM-only. Flow cytometry was used to identify four categories of T-cell memory phenotypes based on the expression of CD45RA and CCR7 as a percentage of total CD4^+^ and CD8^+^ T cells. The T-cell memory phenotypes were defined as naïve (CD45RA^+^CCR7^+^), central memory (CM; CD45RA^−^CCR7^+^), effector memory (EM; CD45RA^−^CCR7^−^), and terminally differentiated effector memory (TEMRA; CD45RA^+^CCR7^−^) (**Figures 1A, B**). Naïve CD4^+^ T cells were significantly decreased in the LTBI-DM compared to the LTBI-only participants (p<0.001), with naïve CD8^+^ T cells being slightly decreased in the same participants (p=0.112) (**Figures 1C, E, D, F**). Additionally, central memory CD4^+^ and CD8^+^ T-cell frequencies were significantly increased in the LTBI-DM compared to the LTBI-only participants [(p=0.002) and (p=0.044)] respectively (**Figures 1C, E, D, F**). Compared to LTBI-only, participants with LTBI-DM had significantly increased effector memory CD4^+^ T cells (p=0.012) (**Figures 1C, E**). No differences were observed for TEMRA CD4^+^ and CD8^+^ T cells.

#### Type 2 DM impairs *Mtb*-specific CD4^+^ and CD8^+^ T activation, exhaustion and degranulation

**Figure 2:**
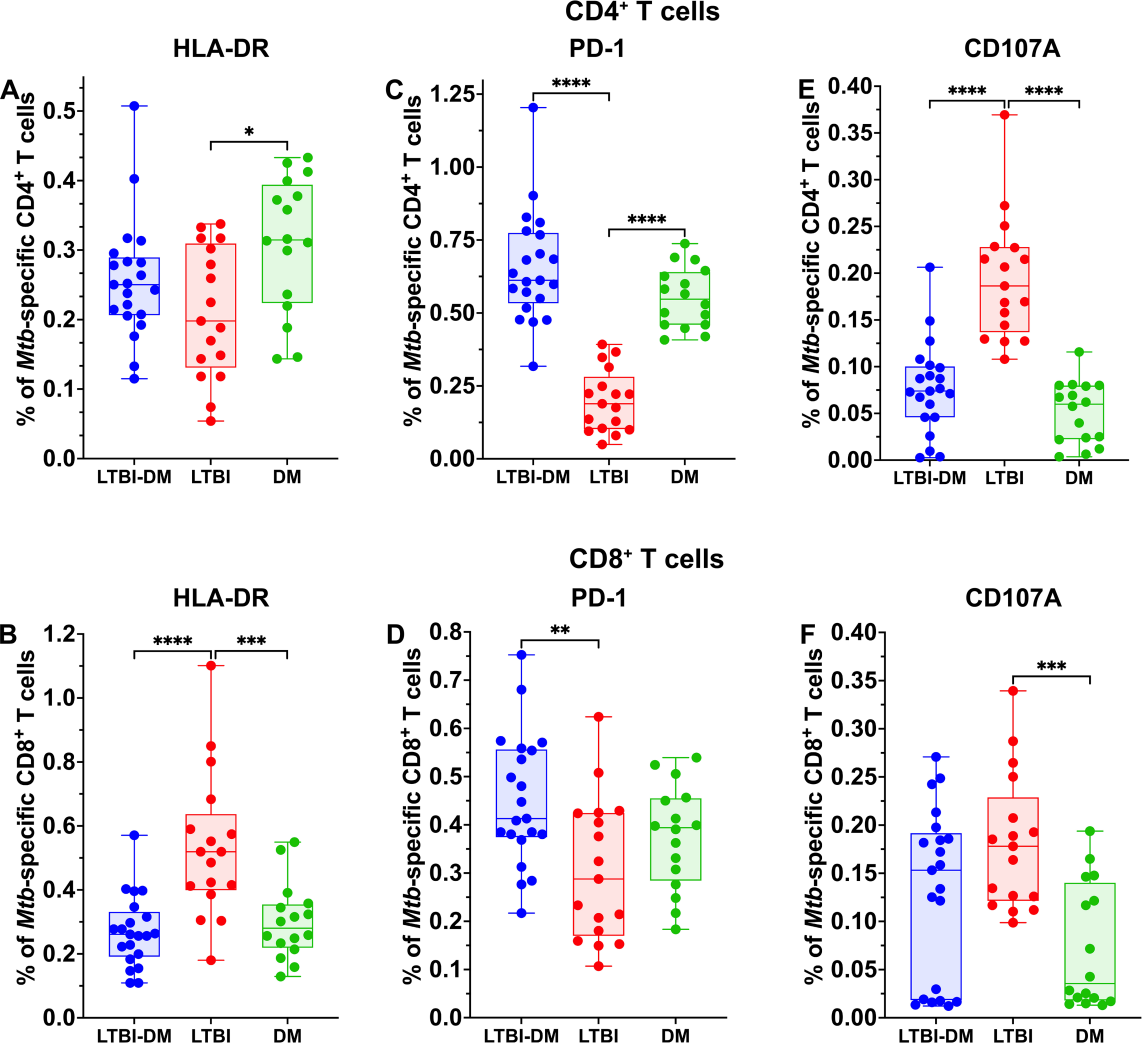
Type 2 DM impairs the HLA-DR, PD-1, and CD107A expression of Mtb-specific CD4+ and CD8+ T cells. The PBCMs were surface stained with HLA-DR and PD-1 antibodies after 18 hours of incubation with ESAT-6 and CFP-10 peptide pools and brefeldin A. **(E, F)** For degranulation analysis of CD4^+^ and CD8^+^ T cells, CD107a was added during stimulation. **(A-D)** Representative plots for HLA-DR and PD-1. Size of participant groups: LTBI-DM (n = 21), LTBI (n = 17), DM (n = 16). Data represent medians and interquartile ranges. The non-parametric Kruskal-Wallis and Mann-Whitney U tests were used to determine the statistical significance between the medians. p< 0.05 (*), p< 0.01 (**), p<0.001 (***) and p< 0.0001 (****). Non-significant p-values were not shown.

HLA-DR, an activation marker, is expressed on several cellular populations, including CD4^+^ and CD8^+^ T cells (**Figures 2A, B**). *Mtb*-specific HLA-DR expression on CD8^+^ T cells was significantly decreased in LTBI-DM (**Figure 2B**) compared to LTBI-only participants (p<0.0001). Interestingly, *Mtb*-specific CD4^+^ and CD8^+^ T-cell PD-1 expression was significantly upregulated in the LTBI-DM compared to the LTBI-only participants [(p<0.0001) and (p=0.007)] respectively (**Figures 2C, D**). PBMCs were stained with CD107a (during incubation) to determine CD107a production. Compared to LTBI-only, participants with LTBI-DM had significantly impaired CD107a production by CD4^+^ T cells (p<0.0001) (**Figure 2E**). Though non-significant, LTBI-DM participants had slightly impaired CD107a production by CD8^+^ T cells compared to the LTBI-only participants (p=0.161) (**Figure 2F**).

#### Type 2 DM impairs the production of *Mtb*-specific Th-1, Th-2 and Th-17 cytokines by CD4^+^ and CD8^+^ T cells

**Figure 3:**
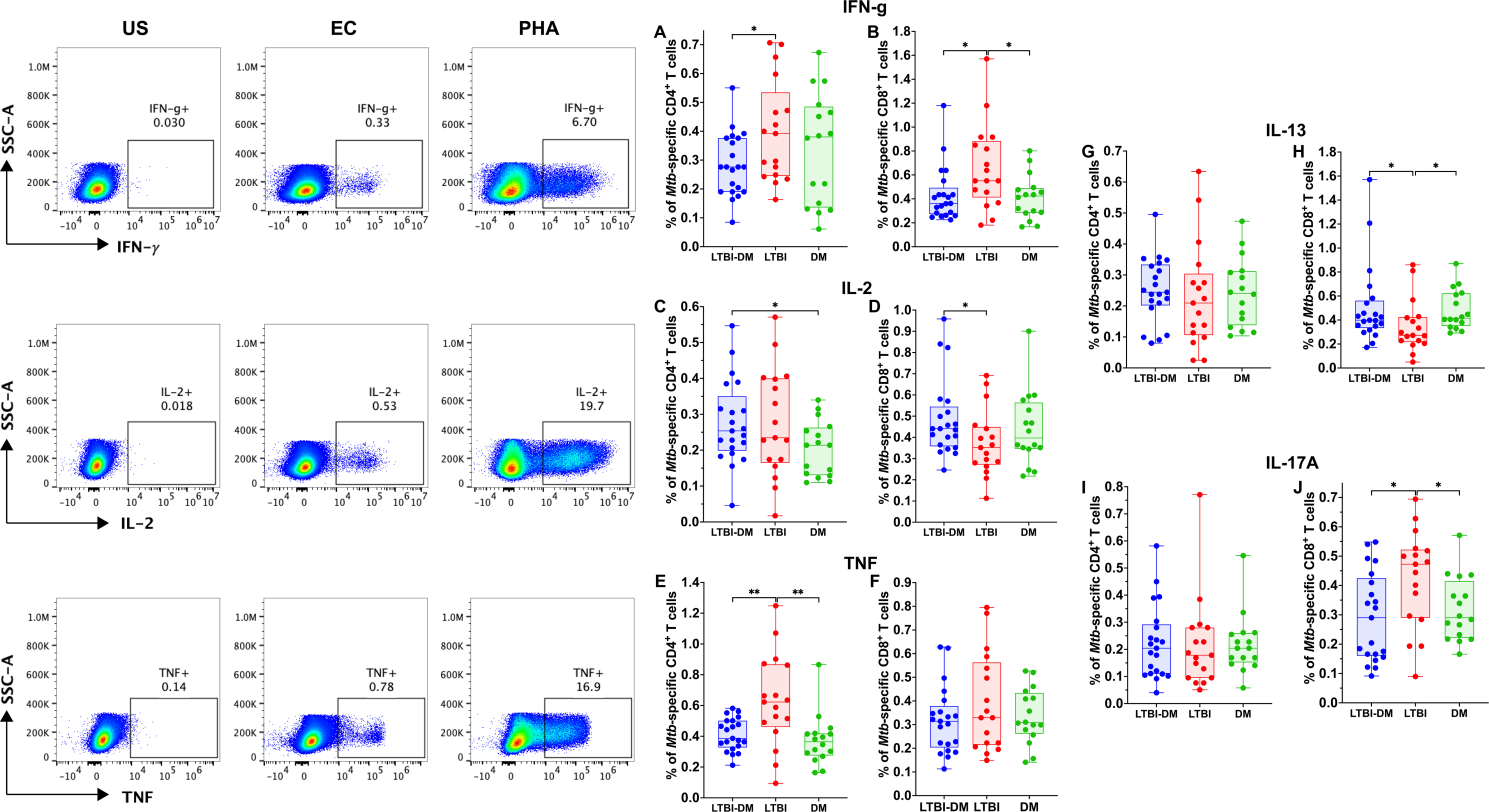
Type 2 DM impairs the production of *Mtb*-specific Th-1, Th-2 and Th-17 cytokines by CD4^+^ and CD8^+^ T cells. The PBMCs were cultured and stimulated for 18 hours with ESAT-6 and CFP-10 peptide pools, brefeldin A, and intracellularly stained for cytokines. **(A-J)** Representative plots for CD4^+^ and CD8^+^ T-cell producing TNF, IFN-γ, IL-2, IL-13, IL-17A cytokines. Size of participant groups: LTBI-DM (n = 21), LTBI (n = 17), DM (n = 16). Data represent medians and interquartile ranges. The non-parametric Kruskal-Wallis and Mann-Whitney U tests were used to determine the statistical significance between the medians. p< 0.05 (*), p< 0.01 (**). Non-significant p-values were not shown.

To determine CD4^+^ and CD8^+^ T-cell functionality in terms of cytokine expression, PBMCs were stained with TNF, IFN-γ, IL-2, IL-13 and IL-17A (intracellularly) (**Figure 3**). Of the Th-1 cytokines, CD4^+^ and CD8^+^ T-cell *Mtb*-specific IFN-γ production was significantly decreased in the LTBI-DM compared to the LTBI-only participants [(p=0.030) and (p=0.016)] respectively (**Figures 3A, B**). Additionally, CD4^+^ T-cell *Mtb*-specific TNF production was significantly decreased in participants with LTBI-DM compared to those with LTBI-only (p=0.004) (**Figure 3E**). Finally, CD8^+^ T-cell *Mtb*-specific IL-13 and IL-17A production were increased and decreased in the LTBI-DM compared to the LTBI-only participants, respectively [(p=0.033) and (0.017)] (**Figures 3H, J**).

#### Type 2 DM impairs dual and mono-functional *Mtb*-specific CD4^+^ and CD8^+^ T-cell responses

**Figure 4:**
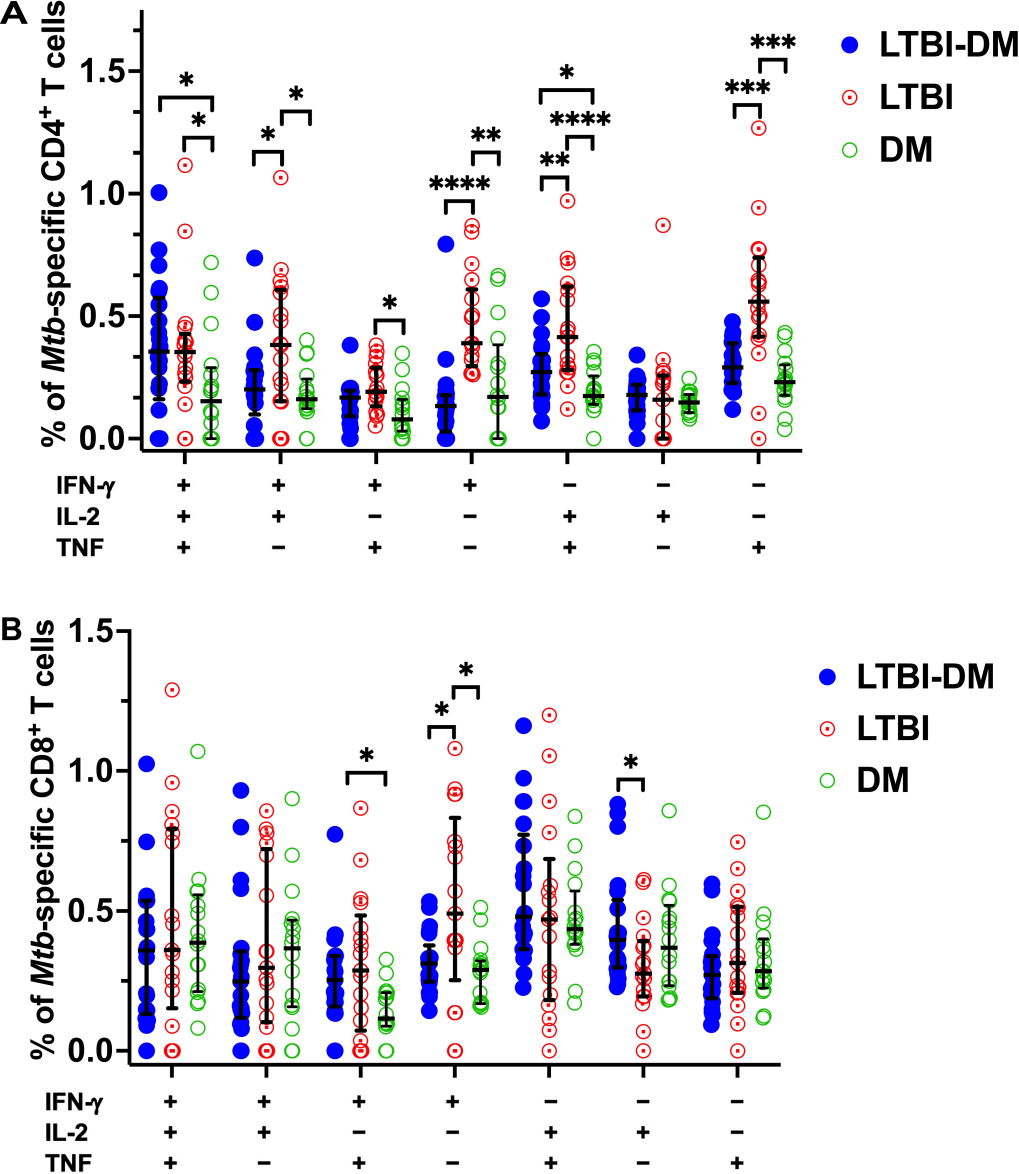
Type 2 DM impairs dual and mono-functional *Mtb*-specific CD4^+^ and CD8^+^ T-cell responses. **(A)** Polyfunctional *Mtb*-specific CD4^+^ T-cell responses. **(B)** Polyfunctional *Mtb*-specific CD8^+^ T-cell responses. The X-axis represents the frequencies of *Mtb-*specific CD4^+^ T cells producing all possible IFN-γ, IL-2 and TNF combinations. Data represent medians and interquartile ranges. The non-parametric Kruskal-Wallis and Mann-Whitney U tests were used to determine the statistical significance between the medians. p< 0.05 (*), p< 0.01 (**), p<0.001 (***) and p< 0.0001 (****).

To further analyse the quality of *Mtb-*specific CD4^+^ and CD8^+^ T-cell responses, we defined the polyfunctional potential of *Mtb*-specific CD4^+^ and CD8^+^ T-cell responses based on their capacity to co-express IFN-γ, IL-2 or TNF by applying the Boolean gating strategy to all samples using FlowJo and subtracting the non-specific polyfunctional responses (**Figure 4**). LTBI-DM participants had significantly lower frequencies of dual-functional IFN-γ^+^IL-2^+^ (p=0.018) and IL-2^+^TNF^+^ (p=0.006) CD4^+^ T cells compared to LTBI-only participants (**Figure 4A**). Additionally, mono-functional IFN-γ^+^ (p<0.0001) and TNF^+^ (p<0.001) CD4^+^ T-cell responses were significantly decreased in participants with LTBI-DM compared to those with LTBI-only (**Figure 4A**). Regarding CD8^+^ T-cell polyfunctionality, only mono-functional IFN-γ^+^ responses decreased significantly in participants with LTBI-DM compared to those with LTBI-only (p=0.033) (**Figure 4B**).

#### Type 2 DM impairs triple, dual, mono-functional *Mtb*-specific central and effector memory CD4^+^ T cell responses

**Figure 5:**
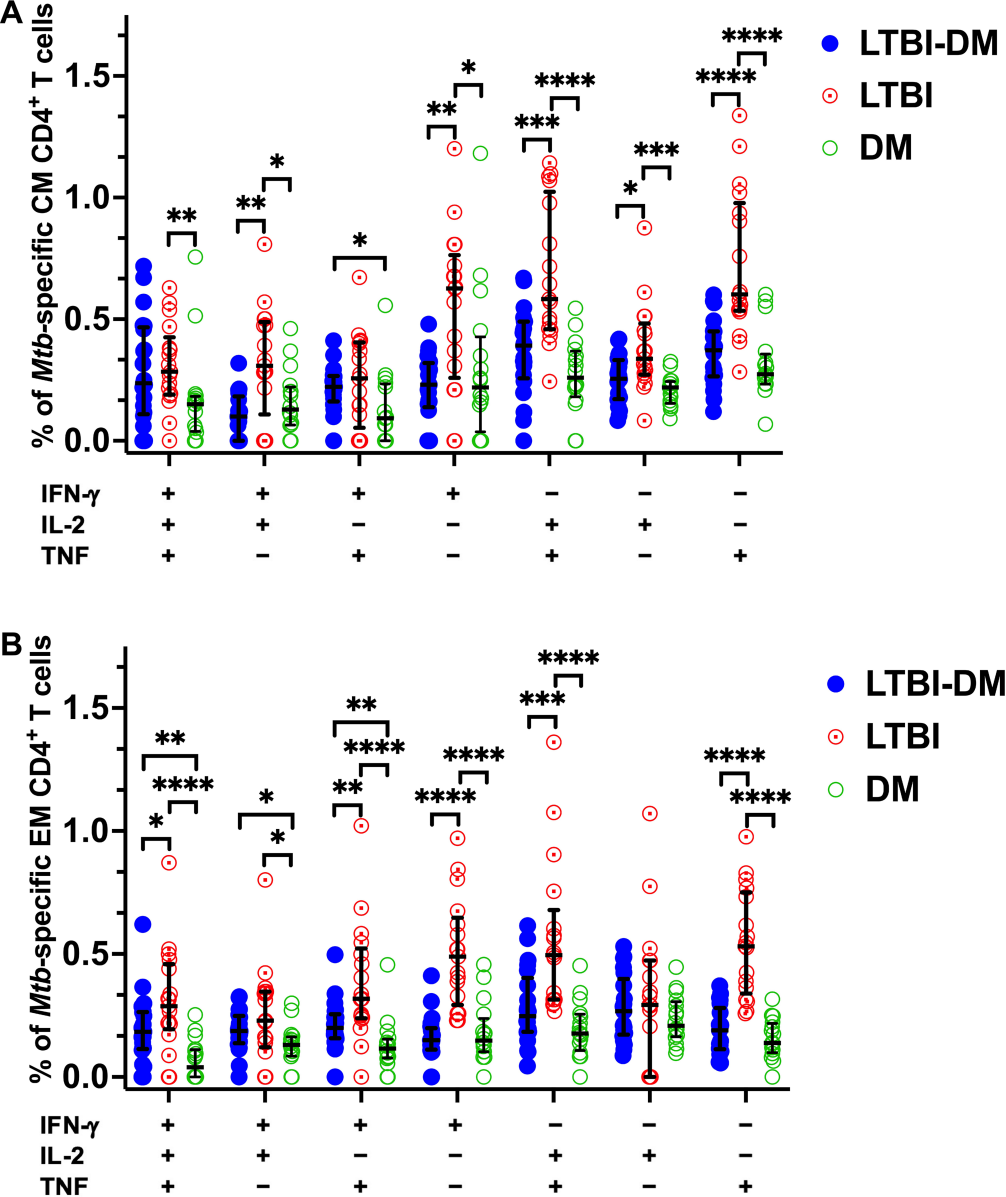
Type 2 DM impairs triple, dual, mono-functional *Mtb*-specific central and effector memory CD4^+^ T-cell responses. **(A)** Polyfunctional *Mtb*-specific central memory CD4^+^ T-cell responses. **(B)** Polyfunctional *Mtb*-specific effector memory CD4^+^ T-cell responses. The X-axis represents the frequencies of *Mtb-*specific central and effector memory CD4^+^ T cells producing all possible combinations of IFN-γ, IL-2 and TNF. Data represent medians and interquartile ranges. Kruskal-Wallis and Mann-Whitney U tests were used to determine the statistical significance between the medians. p< 0.05 (*), p< 0.01 (**), p<0.001 (***) and p< 0.0001 (****). CM, Central memory; EM, Effector memory.

Following on from our previous result, Boolean gating strategy was further applied to all samples’ CD4^+^ T-cell central and effector memory responses to determine their polyfunctional capacity to produce *Mtb*-specific IFN-γ, IL-2 or TNF after non-specific polyfunctional cytokine production subtraction (**Figure 5**). With regards to central memory CD4^+^ T-cell responses, LTBI-DM participants had decreased dual-functional IFN-γ^+^IL-2^+^ (p=0.002) and IL-2^+^TNF^+^ (p<0.001) frequencies compared to LTBI-only participants (**Figure 5A**). Additionally, mono-functional IFN-γ^+^ (p=0.001), IL-2^+^ (p=0.011) and TNF^+^ (p<0.0001) central memory CD4^+^ T-cell responses were significantly decreased in participants with LTBI-DM compared to those with LTBI-only (**Figure 5A**). Regarding effector memory CD4^+^ T-cell responses, LTBI-DM participants had decreased triple functional IFN-γ^+^IL-2^+^TNF^+^ (p=0.033), dual-functional IFN-γ^+^ TNF^+^ (p=0.004) and IL-2^+^TNF^+^ (p<0.001) frequencies compared to LTBI-only participants (**Figure 5B**). Additionally, mono-functional IFN-γ^+^ (p<0.0001) and TNF^+^ (p<0.0001) effector memory CD4^+^ T-cell responses were significantly decreased in participants with LTBI-DM compared to those with LTBI-only (**Figure 5B**).

## Discussion

Immunological dysregulation is one mechanism that accounts for TB susceptibility and severity in DM, but it is not well elucidated and remains poorly characterised. We performed an extended analysis of the memory phenotypes and functional responses of *Mtb*-specific CD4^+^ and CD8^+^ T cells to identify immunological differences between LTBI-DM, LTBI-only and DM-only participants. Our study identified three key points: 1) Type 2 DM alters the memory phenotype of CD4^+^ and CD8^+^ T cells; 2) Type 2 DM impairs T-cell activation and degranulation but promotes T-cell exhaustion; 3) Type 2 DM impairs the CD4^+^ and CD8^+^ T-cell Th1, Th2 and Th17 cytokine responses, as well as the polyfunctional (triple, dual, mono) capacity of the CD4^+^ T-cell, and central and effector memory CD4^+^ T-cell subsets. We showed that type 2 DM is associated with profound impairment of *Mtb*-specific T-cell responses, which could increase TB susceptibility.

This study reveals that naïve CD4^+^ T cells were decreased, whereas the CD4^+^ and CD8^+^ T-cell central and effector memory phenotypes were increased in the LTBI-DM compared to the LTBI-only participants. The reduction in naïve CD4^+^ T cells is similar to a study by Kumar and colleagues, who reported decreased naïve CD4 T cells in active TB with DM participants (25). The decrease indicates a potential compromise towards delayed or insufficient immune responses against *Mtb* reactivation, allowing *Mtb* to potentially proliferate and increase susceptibility to active TB disease (26). The significant increase of central and effector memory T-cell frequencies in LTBI-DM participants implies a shift towards an activated memory T-cell phenotype. Memory T cells are crucial for long-term immune surveillance (27, 28) and rapid response upon re-exposure to *Mtb* (29). This increase may reflect an immune response to chronic *Mtb* stimulation or a compensatory mechanism in response to impaired naïve T-cell function. This could have implications for both TB protection and disease progression, as an increased T-cell memory phenotype could potentially contribute to *Mtb*-related chronic inflammation, resulting in T-cell memory cells with impaired immune function, including exhaustion, activation, homing and cytokine production (30). Type 2 DM orchestrated T-cell memory alteration may potentially decrease the overall robustness of the T-cell memory response, potentially increasing susceptibility to active TB disease.

The functional profiles and fitness of the T cells are significant factors to consider when assessing *Mtb*-specific responses in the face of DM. Our study reports significant upregulation of PD-1 on T cells in the LTBI-DM participants, a consensus to several studies that reported upregulation of PD-1 expression on T cells during *Mtb* infection and active TB disease (15, 16). PD-1 impairs T-cell proliferation during active TB disease (16) and Th1 immune function during *Mycobacterium bovis* BCG vaccination (31). Type 2 DM promoting increased PD-1 expression could have severe implications for other T-cell functional responses, including activation, degranulation and cytokine production. Interestingly, we report that type 2 DM impairs T-cell activation and degranulation. CD8^+^ T-cell HLA-DR expression was decreased in the LTBI-DM participants compared to the LTBI-only group, an association with a lower activation state, and consistent with another human study that reported impaired HLA-DR expression on H37Rv-infected monocyte-derived macrophages of DM patients (32). HLA-DR is an activating receptor that binds and presents antigens to T cells, thereby activating immune responses, including cytokine and cytotoxicity functions to clear *Mtb*-infected cells (33). Its expression has also been characterized with effector T cells (34). The decrease in the CD8^+^ T-cell activation state in the face of DM could impair their cytotoxic functions (33), leading to increased risk for LTBI acquisition and ATB progression. However, our study reports that fewer CD8^+^ (but not CD4^+^) T cells were activated. This needs a cautious interpretation, as TB immune impairment is often related to CD4^+^ T-cell HLA-DR dysfunction (35). Interestingly, HLA-DR expression has previously been described as a biomarker that distinguishes LTBI from ATB (36). Whether HLA-DR expression could be used as a biomarker for identifying and distinguishing TB phenotypes in coincident DM remains to be assessed. In addition, our study reports that type 2 DM is associated with compromised CD4^+^ and CD8^+^ T-cell CD107a, a marker for degranulation and cytotoxicity function (37). Similar results have been reported for which type 2 DM compromises the cytotoxic effects of CD8^+^ T and NK cells during active TB (38). CD4^+^ and CD8^+^ T cells have been reported to kill *Mtb*-infected monocytes directly by perforin and Fas/Fas Ligand independent pathways (39). It is important to note differences in the expression profiles of PD-1 and HLA-DR in LTBI-DM and DM groups. These differences may reflect distinct mechanisms of immune activation in the DM group that are not directly related to *Mtb*-specific immune responses in the LTBI-DM group. PD-1 and HLA-DR can be influenced by various factors, including metabolic dysregulation caused by DM (40, 41). Taken together, impairment of HLA-DR expression and CD107a production by DM could promote heightened *Mtb* replication and increased TB risk.

Next, we assessed the effect of DM on CD4^+^ and CD8^+^ T-cell cytokine production, and we observed marked differences in cytokine expression profiles for IFN-γ, IL-2, TNF, IL-13 and IL-17A. CD4^+^ T-cell IFN-γ and TNF, as well as CD8^+^ T-cell IFN-γ and IL-17A production, were decreased, whereas CD8^+^ T-cell IL-13 production was increased in the LTBI-DM participants compared to LTBI-only participants. CD4^+^ and CD8^+^ T-cell IFN-γ production mediates TB protection by controlling the *Mtb* burden and promoting host survival in mice (7) and humans (8). In addition, T-cell-derived TNF plays a crucial role in the early control of TB infection and promotes the formation of mature granulomas and the activation of infected macrophages in mice (42). Similarly, T-cell IL-17A, a Th17 family cytokine, recruits immune cells to *Mtb*-infected sites by upregulating chemokine expression, thereby contributing to granuloma formation and stability (43, 44). On the contrary, increased production of IL-13 is associated with lung damage and the formation of necrotic lesions in mice, which promotes and is consistent with human TB pathology (45, 46). Impairment of the CD4^+^ and CD8^+^ T-cell cytokine responses by DM in the face of TB infection could promote *Mtb* replication, thus promoting TB pathology.

Lastly, we assessed the effect of DM on combinations of polyfunctional Th1 cytokine co-expression profiles of CD4^+^ and CD8^+^ T cells, as well as CD4^+^ T-cell memory phenotypes. Several studies that have profiled the role of polyfunctional CD4^+^ T cells in producing multiple Th1 cytokines (IFN-γ, IL-2, TNF) during TB infection have associated polyfunctional CD4^+^ T cells with protection against TB (47–51). It is conceivable that polyfunctional T cells are more effective at controlling infection than those producing single cytokines. Whether these can be used as targets for TB vaccination in the face of DM remains to be assessed in more extensive studies. Our study is among the first to evaluate the impact of type 2 DM on CD4^+^ and CD8^+^ T-cell polyfunctionality, as well as the CD4^+^ T-cell central and effector memory polyfunctionality. Interestingly, BCG vaccination in mice and humans has been reported to induce polyfunctional CD4 central and effector memory T cells that confer protective memory immunity against TB in a mice model (11, 12). Our data reveals that DM significantly impairs the dual (IFN-γ^+^IL-2^+^ and IL-2^+^TNF^+^) and mono (IFN-γ^+^ and TNF^+^)-functional capacity of *Mtb*-specific CD4^+^ T cells in the LTBI-DM compared to the LTBI-only participants. Additionally, DM significantly impaired the triple (EM: IFN-γ^+^IL-2^+^TNF^+^), dual (CM: IFN-γ^+^IL-2^+^ and IL-2^+^TNF^+^; EM: IFN-γ^+^ TNF^+^ and IL-2^+^TNF^+^), and mono (CM: IFN-γ^+^, IL-2^+^ and TNF^+^; EM: IFN-γ^+^ and TNF^+^)-functional capacity of the *Mtb*-specific CD4^+^ T-cell central and effector memory responses, contributing to first evidence of DM immune impairment on polyfunctional CD4^+^ T-cell memory responses. The results are consistent with a study by Kumar et al. and colleagues (52) that reported diminished frequencies of dual- and mono-functional CD4^+^ T cells in LTBI-DM participants. Moreover, Kamboj et al. (53) reported improved *Mtb* clearance after restoring dual functional IFN-γ^+^TNF^+^ CD4^+^ T cells, further highlighting the importance of polyfunctional T cells as correlates of TB protection. This study demonstrates DM immune-modulatory effects and impairment of both *Mtb*-specific CD4^+^ T cells and their central and effector memory polyfunctional responses during TB progression. This may promote increased TB disease risk and increase active TB progression.

This study faces limitations, including a limited sample size. It is also important to note that the data generated after *in vitro* culture may not represent what occurs *in vivo*. In addition, HbA1c and other DM-related parameters were not collected for participants in the LTBI-only group as these were from another control group comprised of household contacts of TB index patients (KTB study). As a result, our analysis could not adjust for HbA1c levels across all groups. Hence, there remains a possibility of residual confounding related to diabetes severity, which could influence some of the observed immune differences between groups. Lastly, this focused exclusively on T-cell responses to peptides derived from ESAT6 and CFP10 peptides, representing only a subset of the numerous antigens expressed by *Mtb*. Consequently, the findings related to T-cell responses in this study may not be fully generalizable to the overall T-cell response to *Mtb*.

In summary, this study advances the understanding of immune impairment in the LTBI-DM comorbidity. Type 2 DM impairs the memory phenotype and polyfunctional profiles of *Mtb*-specific CD4^+^ and CD8^+^ T cells, which could influence the LTBI-DM immunopathology towards increased TB disease risk.

## Data Availability

The original contributions presented in the study are included in the article/**Supplementary Material**. Further inquiries can be directed to the corresponding author.

## References

[1] Siddiqui AN Hussain S Siddiqui N Khayyam KU Tabrez S Sharma M. Detrimental association between diabetes and tuberculosis: An unresolved double trouble. Diabetes Metab Syndrome: Clin Res Rev. (2018) 12:1101–7. doi: 10.1016/j.dsx.2018.05.009 29802074

[2] Sun H Saeedi P Karuranga S. IDF Diabetes Atlas: Global, regional and country-level diabetes prevalence estimates for 2021 and projections for 2045. Diabetes Res Clin Prac. (2022) 183:109119. doi: 10.1016/j.diabres.2021.109119 PMC11057359 34879977

[3] World Health Organization. Global tuberculosis report 2023. Geneva: WHO (2023).

[4] Jeon CY Murray MB. Diabetes mellitus increases the risk of active tuberculosis: a systematic review of 13 observational studies. PLoS Med. (2008) 5:e152. doi: 10.1371/journal.pmed.0050152 18630984 PMC2459204

[5] Kibirige D Andia-Biraro I Olum R Adakun S Zawedde-Muyanja S Sekaggya-Wiltshire C. Tuberculosis and diabetes mellitus comorbidity in an adult Ugandan population. BMC Infect Diseases. (2024) 24:242. doi: 10.1186/s12879-024-09111-8 38389045 PMC10885501

[6] Kibirige D Andia-Biraro I Kyazze AP Olum R Bongomin F Nakavuma RM. Burden and associated phenotypic characteristics of tuberculosis infection in adult Africans with diabetes: a systematic review. Sci Rep. (2023) 13:19894. doi: 10.1038/s41598-023-47285-4 37963989 PMC10645877

[7] Green AM DiFazio R Flynn JL. IFN-γ from CD4 T Cells Is Essential for Host Survival and Enhances CD8 T Cell Function during Mycobacterium tuberculosis Infection. J Immunol. (2013) 190:270–7. doi: 10.4049/jimmunol.1200061 PMC3683563 23233724

[8] Rueda CM Marín ND García LF Rojas M. Characterization of CD4 and CD8 T cells producing IFN-γ in human latent and active tuberculosis. Tuberculosis. (2010) 90:346–53. doi: 10.1016/j.tube.2010.09.003 20933471

[9] Lindestam Arlehamn CS Gerasimova A Mele F Henderson R Swann J Greenbaum JA. Memory T Cells in Latent Mycobacterium tuberculosis Infection Are Directed against Three Antigenic Islands and Largely Contained in a CXCR3+CCR6+ Th1 Subset. PLoS Pathogens. (2013) 9:e1003130. doi: 10.1371/journal.ppat.1003130 23358848 PMC3554618

[10] Soares AP Kwong Chung CKC Choice T Hughes EJ Jacobs G van Rensburg EJ. Longitudinal changes in CD4+ T-cell memory responses induced by BCG vaccination of newborns. J Infect Diseases. (2013) 207:1084–94. doi: 10.1093/infdis/jis941 PMC3583271 23293360

[11] Cruz A Torrado E Carmona J Fraga AG Costa P Rodrigues F. BCG vaccination-induced long-lasting control of Mycobacterium tuberculosis correlates with the accumulation of a novel population of CD4+IL-17+TNF+IL-2+ T cells. Vaccine. (2015) 33:85–91. doi: 10.1016/j.vaccine.2014.11.013 25448107

[12] Ancelet LR Aldwell FE Rich FJ Kirman JR. Oral vaccination with lipid-formulated BCG induces a long-lived, multifunctional CD4+ T cell memory immune response. PLoS One. (2012) 7:e45888. doi: 10.1371/journal.pone.0045888 23049885 PMC3457949

[13] Riou C Du Bruyn E Ruzive S Goliath RT Lindestam Arlehamn CS Sette A. Disease extent and anti-tubercular treatment response correlates with Mycobacterium tuberculosis-specific CD4 T-cell phenotype regardless of HIV-1 status. Clin Trans Immunol. (2020) 9:e1176. doi: 10.1002/cti2.1176 PMC7520805 33005414

[14] Silveira-Mattos PS Barreto-Duarte B Vasconcelos B Fukutani KF Vinhaes CL Oliveira-De-Souza D. Differential expression of activation markers by mycobacterium tuberculosis-specific CD4+ T cell distinguishes extrapulmonary from pulmonary tuberculosis and latent infection. Clin Infect Diseases. (2019) 71:1905–11. doi: 10.1093/cid/ciz1070 PMC8463092 31665254

[15] Jurado JO Alvarez IB Pasquinelli V Martínez GJ Quiroga M Abbate E. Programmed death (PD)-1:PD-ligand 1/PD-ligand 2 pathway inhibits T cell effector functions during human tuberculosis1. J Immunol. (2008) 181:116–25. doi: 10.4049/jimmunol.181.1.116 18566376

[16] Shen L Gao Y Liu Y Zhang B Liu Q Wu J. PD-1/PD-L pathway inhibits M.tb-specific CD4+ T-cell functions and phagocytosis of macrophages in active tuberculosis. Sci Rep. (2016) 6:38362. doi: 10.1038/srep38362 27924827 PMC5141449

[17] Scriba TJ Kalsdorf B Abrahams D-A Isaacs F Hofmeister J Black G. Distinct, specific IL-17- and IL-22-producing CD4+ T cell subsets contribute to the human anti-mycobacterial immune response1. J Immunol. (2008) 180:1962–70. doi: 10.4049/jimmunol.180.3.1962 PMC2219462 18209095

[18] Chen X Zhang M Liao M Graner MW Wu C Yang Q. Reduced Th17 response in patients with tuberculosis correlates with IL-6R expression on CD4+ T cells. Am J Respir Crit Care Med. (2010) 181:734–42. doi: 10.1164/rccm.200909-1463OC 20019339

[19] Day CL Abrahams DA Lerumo L Janse van Rensburg E Stone L O’rie T. Functional capacity of mycobacterium tuberculosis-specific T cell responses in humans is associated with mycobacterial load. J Immunol. (2011) 187:2222–32. doi: 10.4049/jimmunol.1101122 PMC3159795 21775682

[20] Harari A Rozot V Enders FB Perreau M Stalder JM Nicod LP. Dominant TNF-α+ Mycobacterium tuberculosis–specific CD4+ T cell responses discriminate between latent infection and active disease. Nat Med. (2011) 17:372–6. doi: 10.1038/nm.2299 PMC6570988 21336285

[21] Kumar NP Sridhar R Banurekha VV Jawahar MS Nutman TB Babu S. Expansion of pathogen-specific T-helper 1 and T-helper 17 cells in pulmonary tuberculosis with coincident type 2 diabetes mellitus. J Infect diseases. (2013) 208:739–48. doi: 10.1093/infdis/jit241 PMC3733509 23715661

[22] Ssekamatte P Nakibuule M Nabatanzi R Egesa M Musubika C Bbuye M. Type 2 diabetes mellitus and latent tuberculosis infection moderately influence innate lymphoid cell immune responses in Uganda. Front Immunol. (2021) 12. doi: 10.3389/fimmu.2021.716819 PMC8432960 34512639

[23] Biraro IA Egesa M Toulza F Levin J Cose S Joloba M. Impact of co-infections and BCG immunisation on immune responses among household contacts of tuberculosis patients in a Ugandan cohort. PloS One. (2014) 9:e111517. doi: 10.1371/journal.pone.0111517 25372043 PMC4221037

[24] American Diabetes Association Professional Practice Committee. Classification and diagnosis of diabetes: standards of medical care in diabetes—2022. Diabetes Care. (2022) 45:S17–38. doi: 10.2337/dc22-S002 34964875

[25] Kumar NP Moideen K Dhakshinraj SD Banurekha VV Nair D Dolla C. Profiling leucocyte subsets in tuberculosis-diabetes co-morbidity. Immunology. (2015) 146:243–50. doi: 10.1111/imm.2015.146.issue-2 PMC4582965 26095067

[26] Liu X Li H Li S Yuan J Pang Y. Maintenance and recall of memory T cell populations against tuberculosis: Implications for vaccine design. Front Immunol. (2023) 14. doi: 10.3389/fimmu.2023.1100741 PMC10102482 37063832

[27] Sallusto F Lenig D Förster R Lipp M Lanzavecchia A. Two subsets of memory T lymphocytes with distinct homing potentials and effector functions. Nature. (1999) 401:708–12. doi: 10.1038/44385 10537110

[28] Masopust D Vezys V Marzo AL Lefrançois L. Preferential localization of effector memory cells in nonlymphoid tissue. Science. (2001) 291:2413–7. doi: 10.1126/science.1058867 11264538

[29] Qin S Chen R Jiang Y Zhu H Chen L Chen Y. Multifunctional T cell response in active pulmonary tuberculosis patients. Int Immunopharmacology. (2021) 99:107898. doi: 10.1016/j.intimp.2021.107898 34333359

[30] Behar SM Carpenter SM Booty MG Barber DL Jayaraman P. Orchestration of pulmonary T cell immunity during Mycobacterium tuberculosis infection: Immunity interruptus. Semin Immunol. (2014) 26:559–77. doi: 10.1016/j.smim.2014.09.003 PMC4250436 25311810

[31] Sakai S Kawamura I Okazaki T Tsuchiya K Uchiyama R Mitsuyama M. PD-1–PD-L1 pathway impairs Th1 immune response in the late stage of infection with Mycobacterium bovis bacillus Calmette–Guérin. Int Immunol. (2010) 22:915–25. doi: 10.1093/intimm/dxq446 21047981

[32] Lopez-Lopez N Martinez AGR Garcia-Hernandez MH Hernandez-Pando R Castañeda-Delgado JE Lugo-Villarino G. Type-2 diabetes alters the basal phenotype of human macrophages and diminishes their capacity to respond, internalise, and control Mycobacterium tuberculosis. Memórias do Instituto Oswaldo Cruz. (2018) 113:e170326. doi: 10.1590/0074-02760170326 29513874 PMC5851047

[33] Yang Y Shi H Zhou Y Zhou Y. Expression of HLA-DR and KLRG1 enhances the cytotoxic potential and cytokine secretion capacity of CD3+ T cells in tuberculosis patients. Int Immunopharmacology. (2024) 133:112115. doi: 10.1016/j.intimp.2024.112115 38652959

[34] Tippalagama R Singhania A Dubelko P Arlehamn CSL Crinklaw A Pomaznoy M. HLA-DR marks recently divided antigen-specific effector CD4 T cells in active tuberculosis patients. J Immunol. (2021) 207:523–33. doi: 10.4049/jimmunol.2100011 PMC8516689 34193602

[35] Ahmed A Adiga V Nayak S Uday Kumar JAJ Dhar C Sahoo PN. Circulating HLA-DR+CD4+ effector memory T cells resistant to CCR5 and PD-L1 mediated suppression compromise regulatory T cell function in tuberculosis. PloS Pathogens. (2018) 14:e1007289. doi: 10.1371/journal.ppat.1007289 30231065 PMC6166982

[36] Luo Y Xue Y Tang G Lin Q Song H Liu W. Combination of HLA-DR on mycobacterium tuberculosis-specific cells and tuberculosis antigen/phytohemagglutinin ratio for discriminating active tuberculosis from latent tuberculosis infection. Front Immunol. (2021) 12:761209. doi: 10.3389/fimmu.2021.761209 34858413 PMC8632229

[37] Aktas E Kucuksezer UC Bilgic S Erten G Deniz G. Relationship between CD107a expression and cytotoxic activity. Cell Immunol. (2009) 254:149–54. doi: 10.1016/j.cellimm.2008.08.007 18835598

[38] Kumar NP Sridhar R Nair D Banurekha VV Nutman TB Babu S. Type 2 diabetes mellitus is associated with altered CD8(+) T and natural killer cell function in pulmonary tuberculosis. Immunology. (2015) 144:677–86. doi: 10.1111/imm.2015.144.issue-4 PMC4368174 25363329

[39] Canaday DH Wilkinson RJ Li Q Harding CV Silver RF Boom WH. CD4+ and CD8+ T cells kill intracellular mycobacterium tuberculosis by a perforin and Fas/Fas ligand-independent mechanism1. J Immunol. (2001) 167:2734–42. doi: 10.4049/jimmunol.167.5.2734 11509617

[40] Febres-Aldana CA Poppiti R Varlotto JM Voland R Zaleski M Sharzehi S. Diabetes mellitus type 2 is associated with increased tumor expression of programmed death-ligand 1 (PD-L1) in surgically resected non-small cell lung cancer—A matched case-control study. Cancer Treat Res Commun. (2020) 23:100170. doi: 10.1016/j.ctarc.2020.100170 32179498

[41] Restrepo BI Twahirwa M Jagannath C. Hyperglycemia and dyslipidemia: Reduced HLA-DR expression in monocyte subpopulations from diabetes patients. Hum Immunol. (2021) 82:124–9. doi: 10.1016/j.humimm.2020.11.005 PMC9381160 33303215

[42] Allie N Grivennikov SI Keeton R Hsu N-J Bourigault M-L Court N. Prominent role for T cell-derived Tumour Necrosis Factor for sustained control of Mycobacterium tuberculosis infection. Sci Rep. (2013) 3:1809. doi: 10.1038/srep01809 23657146 PMC3648802

[43] Okamoto Yoshida Y Umemura M Yahagi A O’Brien RL Ikuta K Kishihara K. Essential role of IL-17A in the formation of a mycobacterial infection-induced granuloma in the lung. J Immunol. (2010) 184:4414–22. doi: 10.4049/jimmunol.0903332 20212094

[44] Umemura M Yahagi A Hamada S Begum MD Watanabe H Kawakami K. IL-17-Mediated Regulation of Innate and Acquired Immune Response against Pulmonary Mycobacterium bovis Bacille Calmette-Guérin Infection1. J Immunol. (2007) 178:3786–96. doi: 10.4049/jimmunol.178.6.3786 17339477

[45] Walter K Kokesch-Himmelreich J Treu A Waldow F Hillemann D Jakobs N. Interleukin-13-overexpressing mice represent an advanced preclinical model for detecting the distribution of antimycobacterial drugs within centrally necrotizing granulomas. Antimicrobial Agents Chemotherapy. (2022) 66:e01588–21. doi: 10.1128/aac.01588-21 PMC9211424 34871095

[46] Heitmann L Abad Dar M Schreiber T Erdmann H Behrends J Mckenzie AN. The IL-13/IL-4Rα axis is involved in tuberculosis-associated pathology. J Pathology. (2014) 234:338–50. doi: 10.1002/path.2014.234.issue-3 PMC4277691 24979482

[47] Smith SG Zelmer A Blitz R Fletcher HA Dockrell HM. Polyfunctional CD4 T-cells correlate with in *vitro* mycobacterial growth inhibition following Mycobacterium bovis BCG-vaccination of infants. Vaccine. (2016) 34:5298–305. doi: 10.1016/j.vaccine.2016.09.002 27622301

[48] Tebruegge M Ritz N Donath S Dutta B Forbes B Clifford V. Mycobacteria-specific mono- and polyfunctional CD4+ T cell profiles in children with latent and active tuberculosis: A prospective proof-of-concept study. Front Immunol. (2019) 10. doi: 10.3389/fimmu.2019.00431 PMC6459895 31024518

[49] Lindenstrøm T Agger EM Korsholm KS Darrah PA Aagaard C Seder RA. Tuberculosis subunit vaccination provides long-term protective immunity characterized by multifunctional CD4 memory T cells1. J Immunol. (2009) 182:8047–55. doi: 10.4049/jimmunol.0801592 19494330

[50] Rakshit S Ahmed A Adiga V Sundararaj BK Sahoo PN Kenneth J. BCG revaccination boosts adaptive polyfunctional Th1/Th17 and innate effectors in IGRA+ and IGRA– Indian adults. JCI Insight. (2019) 4:e130540. doi: 10.1172/jci.insight.130540 31743110 PMC6975271

[51] Scriba TJ Tameris M Mansoor N Smit E van der Merwe L Isaacs F. Correction: Modified vaccinia Ankara-expressing Ag85A, a novel tuberculosis vaccine, is safe in adolescents and children, and induces polyfunctional CD4+ T cells. Eur J Immunol. (2011) 41:1501–. doi: 10.1002/eji.201190030 PMC3044835 20017188

[52] Kumar NP Moideen K George PJ Dolla C Kumaran P Babu S. Coincident diabetes mellitus modulates Th1-, Th2-, and Th17-cell responses in latent tuberculosis in an IL-10- and TGF-β-dependent manner. Eur J Immunol. (2016) 46:390–9. doi: 10.1002/eji.201545973 PMC6340057 26518995

[53] Kamboj D Gupta P Basil MV Mohan A Guleria R Bhatnagar A. Improved Mycobacterium tuberculosis clearance after the restoration of IFN-γ+TNF-α+CD4+T cells: Impact of PD-1 inhibition in active tuberculosis patients. Eur J Immunol. (2020) 50:736–47. doi: 10.1002/eji.201948283 32113187

[54] Ssekamatte P Obondo SJ Crevel Rv Biraro IA. PA-267 Altered mycobacterium tuberculosis (Mtb)-specific T-cell responses in comorbid tuberculosis and type 2 diabetes mellitus. BMJ Global Health. (2023) 8:A55–A6. doi: 10.1136/bmjgh-2023-EDC.136

