# Supplementary figures and images for: Impaired Mycobacterium tuberculosis-specific T-cell memory phenotypes and functional profiles among adults with type 2 diabetes mellitus in Uganda
Source: Front Immunol. 2024 Oct 4;15:1480739. doi: 10.3389/fimmu.2024.1480739 (PMC11486641; doi:10.3389/fimmu.2024.1480739)

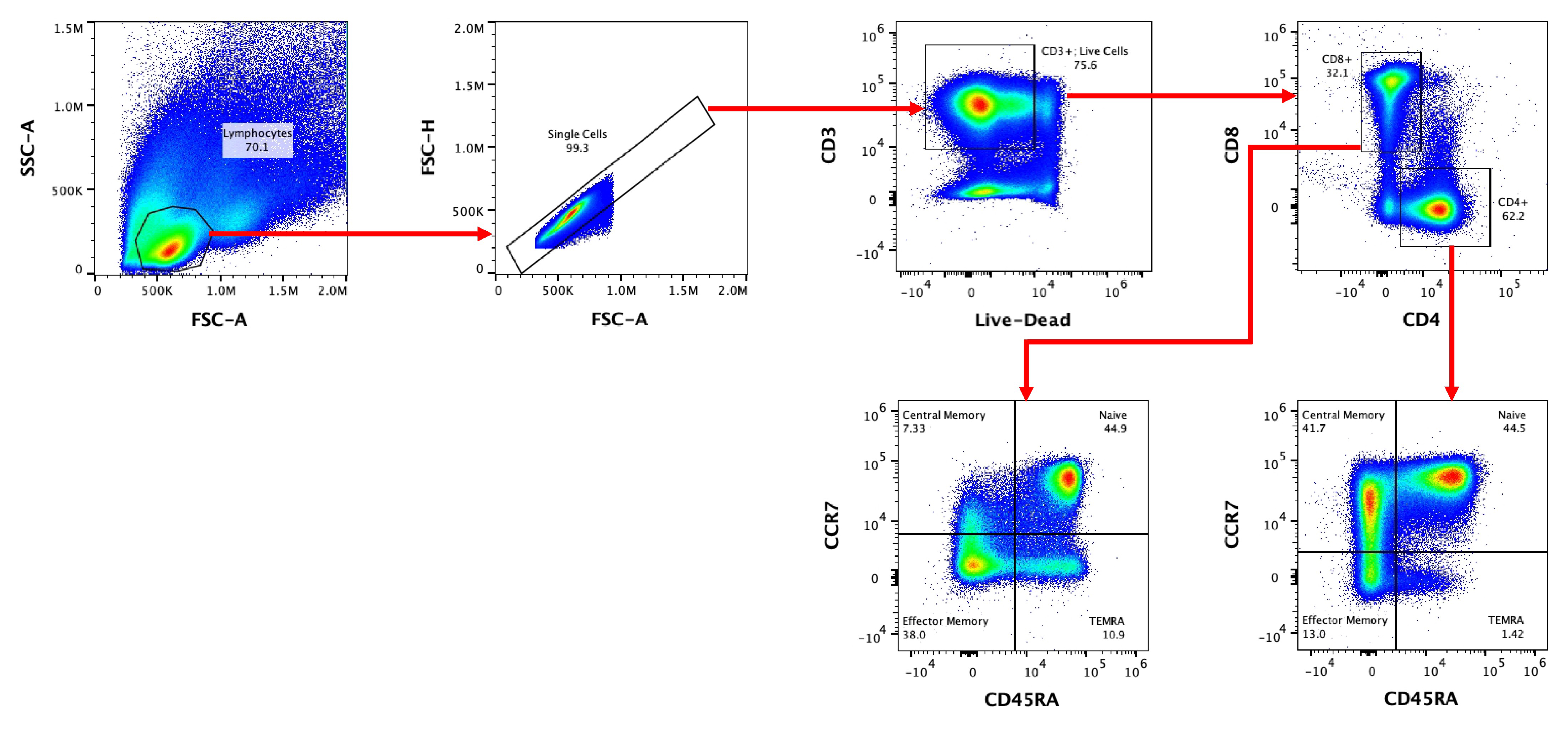

Supplement: Supplementary file 1 [file Image1.jpeg]
