# Supplementary material for: Impaired Mycobacterium tuberculosis-specific T-cell memory phenotypes and functional profiles among adults with type 2 diabetes mellitus in Uganda
Source: Front Immunol. 2024 Oct 4;15:1480739. doi: 10.3389/fimmu.2024.1480739 (PMC11486641; doi:10.3389/fimmu.2024.1480739)
Supplement: Supplementary file 2 [file Table1.docx]

**Supplementary information**

**Supplementary Figure S1: T-cell gating strategy.**

After cell surface and intracellular cytokine staining, single cells were acquired on the CytoFlex LX flow cytometer. All flow cytometry data were analysed using FlowJo (v.10.10.0) for Mac. Gating was standardised and set using Fluorescence Minus One (FMO) controls and compensation controls to correct for spectral overlap. To identify *Mtb-*specific CD4^+^ and CD8^+^ T cells phenotype and function responses, we first gated on single cells, followed by lymphocytes, then CD3^+^ gate in combination with live cells. T-cell populations were identified with traditional gates on CD4 and CD8. T-cell memory responses were then identified based on CD45RA and CCR7 gates. Functional markers (IFN-γ, IL-2, TNF, IL-13, IL-17A, CD107a, HLA-DR, PD-1, BCL-2) were then gated independently on CD3^+^CD4^+^ and CD3^+^CD8^+^ T cells.

**Supplementary Table S1: Flow cytometry panel**

| Role | Marker | Fluorochrome | Clone | Catalogue No. | Manufacturer |
| --- | --- | --- | --- | --- | --- |
| Cell viability | Live Dead | Zombie aqua | N/A | 423101 | BioLegend |
| Lineage | CD3 | FITC | UCHT1 | 300406 | BioLegend |
|  | CD4 | PerCP-Cyanine5.5 | A161A1 | 357414 | BioLegend |
|  | CD8 | BV650 | SK1 | 344730 | BioLegend |
| Memory | CD45RA | APC-Cy7 | HI100 | 304128 | BioLegend |
|  | CCR7 | PE-CF594 | 2-L1-A | 566769 | BD Biosciences |
| Activation | HLA-DR | PE-Fire 640 | L243 | 307676 | BioLegend |
| Apoptosis | Bcl-2 | BV421 | 100 | 658709 | BioLegend |
| Exhaustion | PD-1 | BV785 | EH12.2H7 | 329930 | BioLegend |
| Degranulation | CD107a | BV605 | H4A3 | 328634 | BioLegend |
| Intracellular cytokines | IFN-𝛾 | PE-Cy7 | 4S.B3 | 502528 | BioLegend |
|  | TNF | APC | MAb11 | 502912 | BioLegend |
|  | IL-2 | PE | MQ1-17H12 | 500307 | BioLegend |
|  | IL-13 | AF350 | 32116 | FAB213U-100UG | R&D Systems |
|  | IL-17A | APC-R700 | N49-653 | 565163 | BD Biosciences |
